# In-person 1-day cognitive behavioral therapy-based workshops for postpartum depression: a randomized controlled trial

**DOI:** 10.1017/S0033291723000454

**Published:** 2023-10

**Authors:** Ryan J. Van Lieshout, Haley Layton, Calan D. Savoy, Feng Xie, June S. L. Brown, Kathryn Huh, Peter J. Bieling, David L. Streiner, Mark A. Ferro, Erika Haber-Evans

**Affiliations:** 1 Department of Psychiatry and Behavioural Neurosciences, McMaster University, Hamilton, Ontario, Canada; 2 Health Research Methodology Graduate Program, McMaster University, Hamilton, Ontario, Canada; 3 Department of Health Research Methods, Evidence and Impact, McMaster University, Hamilton, Ontario, Canada; 4 Department of Psychology, Institute of Psychiatry, Psychology and Neuroscience, King's College London, London, UK; 5 Faculty of Health Sciences, McMaster University, Hamilton, Ontario, Canada; 6 School of Public Health and Health Systems, University of Waterloo, Waterloo, Ontario, Canada

**Keywords:** Anxiety, brief, cognitive behavioral therapy, cost effectiveness, depression, mental disorders, postpartum, psychotherapy, public health, randomized controlled trial, workshop

## Abstract

**Background:**

Postpartum depression (PPD) affects up to one in five mothers and birthing parents, yet as few as 10% access evidence-based treatment. One-day cognitive behavioral therapy (CBT)-based workshops for PPD have the potential to reach large numbers of sufferers and be integrated into stepped models of care.

**Methods:**

This randomized controlled trial of 461 mothers and birthing parents in Ontario, Canada with Edinburgh Postnatal Depression Scale (EPDS) scores ⩾10, age ⩾18 years, and an infant <12 months of age compared the effects of a 1-day CBT-based workshop plus treatment as usual (TAU; i.e. care from any provider(s) they wished) to TAU alone at 12-weeks post-intervention on PPD, anxiety, the mother–infant relationship, offspring behavior, health-related quality of life, and cost-effectiveness. Data were collected via REDCap.

**Results:**

Workshops led to meaningful reductions in EPDS scores (*m* = 15.77 to 11.22; *b* = −4.6, *p* < 0.01) and were associated with three times higher odds of a clinically significant decrease in PPD [odds ratio (OR) 3.00, 95% confidence interval (CI) 1.93–4.67]. Anxiety also decreased and participants had three times the odds of clinically significant improvement (OR 3.20, 95% CI 2.03–5.04). Participants reported improvements in mother–infant bonding, infant-focused rejection and anger, and effortful control in their toddlers. The workshop plus TAU achieved similar quality-adjusted life-years at lower costs than TAU alone.

**Conclusions:**

One-day CBT-based workshops for PPD can lead to improvements in depression, anxiety, and the mother–infant relationship and are cost-saving. This intervention could represent a perinatal-specific option that can treat larger numbers of individuals and be integrated into stepped care approaches at reasonable cost.

Postpartum depression (PPD) is among the most common complications of delivery, affecting as many as one in five mothers and birthing parents (Gavin et al., 2005; Lanes, Kuk, & Tamim, 2011). It is associated with an increased risk of future depressive episodes (Slomian, Honvo, Emonts, Reginster, & Bruyère, 2019), and more cognitive, emotional, and behavioral problems in offspring (Goodman & Gotlib, 1999; Murray et al., 2011; Netsi et al., 2018). When those with elevated levels of depressive symptoms are counted, as many as one in three individuals are affected (Meaney, 2018). Those with subsyndromal symptom levels also have poor outcomes as they often fail to seek treatment and are ineligible for clinical services, though many have parenting and offspring outcomes similar to those with formal major depressive disorder (MDD) diagnoses (Holopainen, 2002; Lovejoy, Graczyk, O'Hare, & Neuman, 2000).

While treating PPD can reduce its adverse effects (Krzeczkowski, Schmidt, & Van Lieshout, 2021; Letourneau, Dennis, Cosic, & Linder, 2017), timely and accessible interventions are essential for optimizing outcomes. Healthcare systems are poorly equipped to treat problems requiring urgent psychotherapy like PPD, and as few as one in 10 sufferers receive evidence-based care (Bowen, Bowen, Butt, Rahman, & Muhajarine, 2012; Cox, Sowa, Meltzer-Brody, & Gaynes, 2016). Barriers to treatment include a lack of time and preferences for psychotherapy over medication (Boath, Bradley, & Henshaw, 2004; Byatt, Simas, Lundquist, Johnson, & Ziedonis, 2012; Goodman, 2009; Jones, 2019).

Clinical practice guidelines recommend evidence-based psychotherapies (e.g. cognitive behavioral therapy (CBT)) for the vast majority of those with PPD (MacQueen et al., 2016; NICE, 2020). However, psychological treatments suffer from serious problems of capacity and waitlists can be long. To address this, there needs to be either a massive increase in the number of therapists or substantial changes to the way we treat PPD.

Even though international practice guidelines suggest that those with PPD should be a priority population for psychotherapy access (NICE, 2020), virtually no low-intensity interventions have proven effective in this population. While standard self-help approaches could be effective in those with PPD (Trevillion et al., 2020), they may not be ideally suited for this group. Many mothers and birthing parents also lack the time to engage in self-help exercises which are quite burdensome when added to existing role demands. Moreover, despite their convenience and ease of use, mobile applications (apps) have not proven clinically effective for PPD (Tsai et al., 2022).

One low-intensity approach to treating PPD is 1-day psychotherapy workshops. The delivery of psychotherapy in large groups (i.e. up to 30 participants) (Horrell et al., 2014; Yunus, Musiat, & Brown, 2019) may be capable of addressing the needs of those with PPD, as well as treating it on the scale required to address its prevalence. These 1-day interventions contain the core content of more comprehensive, evidence-based treatments, but their brevity and scalability make them easier to disseminate.

During the COVID-19 pandemic, a large (*n* = 403) randomized controlled trial (RCT) of 1-day CBT-based workshops for PPD delivered online led to reductions in PPD and anxiety, as well as improvements in the mother–infant relationship and infant temperament (Van Lieshout et al., 2021). However, some participants expressed a preference for in-person workshops citing difficulties participating online while minding an infant, concerns about privacy, and the benefits of face-to-face social support. The effectiveness of these workshops outside of the pandemic context also remains unclear, their impact on other children in the home is not well understood, and despite their efficiency, their cost-effectiveness is not known.

A few RCTs of treatments for PPD that have examined cost-effectiveness suggest that effective interventions are usually more expensive than treatment as usual (TAU). One trial of telephone-based peer-support showed that it was more effective than TAU and cost-effective (with an incremental cost-effectiveness ratio (ICER) of CAD$ 10 009), but the cost was higher in the intervention group (Dukhovny et al., 2013). Similarly, a facilitated self-help-based program for PPD was more effective but more costly than TAU at £13 324 per quality-adjusted life-year (QALY) (National Collaborating Centre for Mental Health, 2014). A probabilistic treatment model based on a National Institute for Health and Care Excellence (NICE) guideline found that structured psychological therapy was cost effective compared to TAU at an ICER of £17 481 (Hewitt et al., 2009; NICE, 2020) which is just below the cost-effectiveness threshold of less than £20 000 recommended by NICE (2013). Given the potential scalability of 1-day CBT-based workshops for PPD, understanding its cost-effectiveness will help determine their potential to treat PPD.

Given this background, we examined the effectiveness of in-person 1-day CBT-based workshops for PPD at improving depression, anxiety, the mother–infant relationship, and offspring behavior, as well as health-related quality of life and cost-effectiveness.

## Method

### Trial design and procedures

This parallel-group, multi-site, RCT took place in Ontario, Canada from 17 July 2018 to 28 February 2020, recruiting from seven geographic regions representing most of the provinces of Ontario's local health integration networks: Elgin county, Simcoe county, Prince Edward county, the city of Toronto, Huron county, Kitchener-Waterloo, and Halton regions. Community organizations, midwives, and public health units in these regions assisted with recruitment and provided workshop sites.

Participants in each region were centrally randomized in a 1:1 ratio to the experimental or control group. Individuals assigned to the experimental group received TAU and participated in the 1-day workshop. Participants in the control group also received TAU but were put on a waitlist to receive the CBT workshop 12 weeks later. In Ontario, healthcare is universally available, so TAU could involve care from a physician, nurse practitioner, and/or midwife, as well as pharmacotherapy and/or psychotherapy from a provincially funded program. They could also utilize the services of any private therapist.

Centralized randomization that used 77 blocks of size of four, six, and eight was used to allocate participants to experimental and control arms. The randomization scheme was created in R version 3.4.3 (R Core Team, n.d.) and implemented by the study coordinator in REDCap (Harris et al., 2009), enabling concealment of allocation sequence until group assignment. Staff making reminder calls and/or collecting data and the data analyst were unaware of participant group status. The study was approved by the Hamilton Integrated Research Ethics Board and was registered (ClinicalTrials.gov: NCT03654261).

### Participants

Participants could self-refer after seeing social media advertisements or be referred by staff at a partnering organization. They had to be ⩾18 years old, have an infant <12 months of age, live in a recruitment region, and have an Edinburgh Postnatal Depression Scale (EPDS) score ⩾10. This cut-off is commonly used in clinical and research settings to identify those who may have PPD (Gavin et al., 2005; Lanes et al., 2011) and was chosen to maximize public health relevance [30% of mothers/birthing parents have these symptom levels (Meaney, 2018)]. Participants were also fluent in written and spoken English.

### Intervention

Workshops were delivered by one psychiatrist or one registered psychotherapist randomly assigned to workshop dates. Their training involved review of the participant manual and workshop script. Each delivered one workshop and received feedback from the other prior to delivering study workshops. Workshops were held in community settings such as libraries and community centers, and free childminding was provided.

Each interactive workshop was run from 0900 to 1600 and consisted of four modules containing didactic teaching, group exercises/discussion, and role plays. The first reviewed PPD etiology with an emphasis on cognitive factors, and the second focused on cognitive skills including cognitive restructuring. The third built behavioral skills such as problem solving, behavioral activation, and assertiveness, and the fourth provided an opportunity for action planning. Workshops were based on previous work by Brown and colleagues, but adapted for PPD (Brown, Cochrane, & Hancox, 2000). Participants received a printed copy of the workshop manual.

### Data collection and outcome measures

Data were collected from participants using REDCap (Harris et al., 2009) at baseline (T1) and 12 weeks later (T2). At enrollment, participants self-reported their sociodemographic characteristics (age, infant age, ethnicity, marital status, educational attainment, household income, antidepressant use, and past counseling).

We chose to assess our T2 outcomes at 12 weeks post-treatment because this corresponds to one of the most common post-treatment endpoints used in RCTs of gold-standard psychotherapies like interpersonal therapy (IPT) and CBT. This was intended to help determine if this very brief treatment could produce benefits over comparable periods of time of these well-established treatments. It was felt this could assist patients and clinicians determine if this briefer treatment could be effective and applicable to them.

The primary outcome (PPD) was assessed using the EPDS, a 10-item measure of symptoms of depression during the previous 7 days (Cox, Holden, & Sagovsky, 1987). Each item is scored on a 4-point scale (0–3) with higher scores indicating worse depressive symptoms. Based on Jacobson and Truax's reliable change index (1991), and consistent with the work of others (Affonso, De, Horowitz, & Mayberry, 2000; Matthey, 2004), we defined an EPDS score change of at ⩾4 points as indicative of clinically significant change in PPD.

Secondary outcomes included anxiety, mother–infant bonding, infant temperament, behavior of older children, and cost-effectiveness. The 7-item Generalized Anxiety Disorder Questionnaire (GAD-7) measured anxiety and contains seven items scored on a 0–3-point scale, with higher scores indicating worse anxiety (Spitzer, Kroenke, Williams, & Löwe, 2006). The GAD-7 has been validated for use in postpartum samples (Simpson, Glazer, Michalski, Steiner, & Frey, 2014). In accordance with previous research, ⩾4 point change in GAD-7 score was defined as clinically significant (Toussaint et al., 2020).

The Postpartum Bonding Questionnaire (PBQ) assessed the mother–infant relationship. Its 25-items are scored on a 5-point scale (0–5), and contains four subscales: impaired bonding, rejection and pathological anger toward the infant, infant focused anxiety, and incipient abuse (Brockington, Fraser, & Wilson, 2006). The incipient abuse scale was not examined due to past performance issues (Klier, 2006).

Infant temperament was rated by mothers with the Infant Behavior Questionnaire-Revised Very-Short Form (IBQ-R), a 37-item measure of infant temperament over the past week (Putnam, Helbig, Gartstein, Rothbart, & Leerkes, 2014). Items are scored on a 7-point scale and address three factors: positive affectivity/surgency, negative emotionality, and orienting/regulatory capacity.

Participants also rated the behavior of their 12–36-month-old children (when present) using the Early Childhood Behavior Questionnaire-Short Form (ECBQ), a 107-item measure of temperament comprised of surgency/extraversion, negative affectivity, and effortful control (Putnam, Gartstein, & Rothbart, 2006). Children older than 36 months were rated using the Strength and Difficulties Questionnaire (SDQ), a 25-item measure of child behavior consisting of five scales (emotional, conduct, hyperactivity/inattention, peer relationship problems, and prosocial behavior). The total problems scale, consisting of the first four scales above, was used here (He, Burstein, Schmitz, & Merikangas, 2013).

Participants reported their use of health resources on a scale created using the Canadian Community Health Survey and the Service Use and Resources Form, and adapted for the postpartum period (Government of Canada, 2007). Data on service usage including hospital, physician, and other healthcare provider visits were collected, as were those pertaining to diagnostic tests and medication usage. The costs of workshop delivery were added to healthcare costs determined using a public payer perspective. Service costs were determined primarily by using the Province of Ontario's standardized Schedule of Benefits and its Schedule of Facility Fees. When those were not available, recommended hourly averages reported by the profession's official Ontario association were used (e.g. for physiotherapists, occupational therapists, etc.). The costs of public health programs were averaged based on provincial funding in 2020 (online Supplementary Table S1). The costs of medications were calculated using the Ontario Drug Benefit Formulary.

The EuroQol Group's EQ-5D-3L is a preference-based health status questionnaire comprised of five domains: mobility, self-care, usual activities, pain/discomfort, and anxiety/depression. Each domain is measured through three-level response options indicating no, some, and extreme problems (van Dongen et al., 2021). This study utilized a Canadian value set (Bansback, Tsuchiya, Brazier, & Anis, 2012). These index values were used to compute QALYs to compare the difference in effect between the intervention and control groups (Petrou & Gray, 2011). The EQ-5D-3L can also be scored as a 100-point Visual Analog Scale (VAS), providing a holistic summary of health status across the EQ-5D's five domains (Whynes & the TOMBOLA Group, 2008). The VAS is reported as an outcome measure alongside the core results of this study.

### Statistical analyses

Demographic data were compared using *t* tests and χ^2^ tests for continuous and dichotomous variables, respectively.

*A priori* sample size estimation suggested that a total of 476 participants would provide us with adequate statistical power to detect a medium effect size (*d* = 0.3) change in our primary outcome (EPDS) between groups (a group × time interaction). The power calculation used two-tailed statistical significance (*α* = 0.05) and 1 − *β* = 0.90. This estimation assumes a linear mixed model (LMM) applying a first-order autoregressive error covariance structure. This sample size estimate also presumed a 10% attrition rate between T1 and T2. Because of the COVID-19 pandemic, recruitment was stopped when 461 participants had been recruited. However, this sample size was still estimated to be capable of detecting significant effect sizes of similar magnitude (*d* ~ 0.33). Sample size and power estimations were calculated using RMASS (Bhaumik et al., 2008; Hedeker, Gibbons, & Waternaux, 1999; Roy, Bhaumik, Aryal, & Gibbons, 2007).

To investigate the effectiveness of 1-day CBT workshops on continuous outcomes, we utilized LMMs with restricted maximum-likelihood estimation. A two-level hierarchy was employed in which outcomes at each timepoint (pre- and post-workshop) were nested within participant (split by group), allowing us to assess the effect of the intervention over time between experimental and control groups. A random-effects intercept was also included in the model controlling for unobserved heterogeneity at the level of the individual participant. These models also included workshop facilitator as a fixed effect covariate to control for individual differences in intervention effectiveness that may exist between workshop leaders. For outcome measures that revealed a significant group × time interaction, models stratified by group were employed to identify the magnitude of change in outcome measure score following workshop completion by the experimental group. Cohen's *d* defined the effect size of the change in mean scale score between baseline and follow-up. Finally, logistic regression was used to compare the odds of participants experiencing a ⩾4-point decrease in EPDS and GAD-7 scores (indicative of clinically significant improvement) between T1 and T2. Analyses were performed in SPSS 24 (IBM SPSS Statistics).

In conjunction with the QALY values calculated using the EQ-5D-3L Canadian index scores, healthcare cost data over the 12-week study period were used to calculate the ICER, comparing the cost-effectiveness of the workshop plus TAU *v.* TAU alone. In keeping with the intent-to-treat approach and given the limitations of imputing missing QALY and service use data, complete case analysis was employed. However, non-parametric bootstrapping was applied to the complete cases to estimate the uncertainty of the computed ICER across 1000 bootstrapped simulations. Finally, a cost-effectiveness acceptability curve (CEAC) was derived from the bootstrapped data, illustrating the probability of cost-effectiveness across a range of willingness-to-pay values per QALY gained.

## Results

**Fig. 1.**
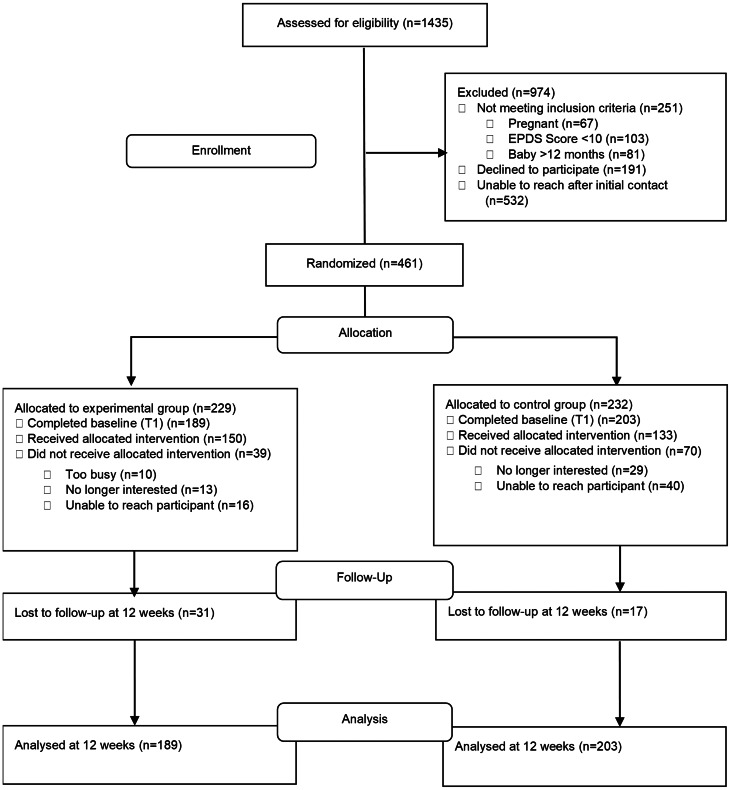
Flowchart of participants through the trial.

A total of 1435 participants were assessed for eligibility and 461 were randomized to experimental (*n* = 229) or control (*n* = 232) groups. Sixty-nine participants declined to participate prior to T1 data collection, so 392 (experimental *n* = 189, control *n* = 203) completed baseline (T1) measures (Fig. 1).

The majority of participants self-referred to the study after seeing information on social media (56%), on a partnering agency's website (5%), or after hearing about the study from family/friends (4%). Others (15%) were referred by healthcare providers including public health nurses, midwives, and/or family doctors. Remaining referral sources included community groups and early years centers.

In total, 33 workshops were run in regions across southern Ontario including Elgin county, Simcoe county, Prince Edward county, the city of Toronto, Huron county, Kitchener-Waterloo, and Halton regions. Workshops were delivered by a registered psychotherapist (*n* = 16) or a psychiatrist (*n* = 17). The baseline characteristics of experimental and control group participants are given in Table 1. There were no statistically significant differences between groups. A total of 48 (12%) participants were lost to follow-up between T1 and T2, of which 31 were in the experimental group and 17 in the waitlist control group (χ^2^ = 5.87, *p* = 0.015). Other than group assignment, no other baseline characteristics predicted dropout. At T1, 37 experimental and 44 control participants completed the ECBQ for their eligible toddlers, and 52 children of those in the experimental group and 55 control participants had children who were age-eligible for SDQ completion.

**Table 1.** Baseline characteristics of experimental and control participants

Demographics	Experimental group	Control group	*t*/χ^2^	*p*
*N*	189	203		
Ethnicity (% white)	134 (69%)	107 (60%)	3.26	0.07
Latino/Hispanic	3 (2%)	11 (6%)		
Middle Eastern	6 (3%)	3 (2%)		
African	2 (1%)	4 (2%)		
Caribbean	5 (3%)	10 (6%)		
South Asian	19 (10%)	11 (6%)		
East Asian	9 (5%)	14 (8%)		
Mixed	8 (4%)	11 (6%)		
Other	9 (5%)	8 (5%)		
Age, years (s.d.)	31.6 (5.2)	32.3 (4.3)	1.39	0.17
Range, years	20–47	19–41		
Infant age, months (s.d.)	5.2 (3.6)	5.6 (3.6)	0.99	0.32
Avg. number of children (s.d.)	1.5 (0.8)	1.5 (0.9)	0.08	0.93
Marital status (% married/common law)	55 (90%)	50 (96%)	2.2	0.53
Years of education	15.6 (2.1)	15.5 (2.1)	0.24	0.81
Household income (CAD)	$80 016 ($17 540)	$82 021 ($19 764)	0.22	0.82
Prior use of talk therapy	79 (44%)	78 (40%)	0.72	0.4
Use of psychotropic medication	16 (8%)	21 (10%)	0.4	0.52
EPDS Baseline score	15.8 (5.9)	15.1 (4.9)	1.3	0.2
GAD-7 Baseline score	11.4 (4.8)	11.4 (4.9)	0.08	0.93
PBQ-IB	13.04 (8.22)	11.79 (6.82)	0.76	0.44
PBQ-RPA	7.05 (5.38)	5.91 (4.45)	1.57	0.12
PBQ-IFA	3.52 (3.71)	3.61 (2.99)	0.65	0.52
IBQ-R-SUR	4.28 (1.31)	4.36 (1.08)	1.13	0.26
IBQ-R-EC	5.14 (1.04)	5.24 (0.87)	0.73	0.47
IBQ-R-NA	3.73 (1.36)	3.82 (1.13)	1.04	0.30
ECBQ-SUR	5.4 (3.26)	5.24 (2.55)	0.14	0.88
ECBQ-NEG	3.31 (2.39)	2.94 (1.88)	1.45	0.15
ECBQ-EC	4.70 (2.56)	5.04 (2.01)	2.00	0.05
SDQ TOTAL	11.20 (11.88)	10.16 (9.8)	0.84	0.40
EQ-5D-VAS	56.46 (21.96)	56.99 (18.72)	0.33	0.74

s.d., standard deviation; CAD, Canadian dollars.

After adjusting for the fixed effect of workshop facilitator, statistically significant group × time interactions predicted EPDS, GAD-7, PBQ-IB, PBQ-RPA, PBQ-IFA, ECBQ-EC, and EQ-5D-3L scores (Table 2). After stratifying by group, experimental group participants reported statistically significant decreases in EPDS scores from T1 to T2, with scores decreasing from 15.77 pre-treatment to 11.22 post-treatment (*p* < 0.01, Cohen's *d* = 0.62). Statistically significant decreases in GAD-7 scores 11.36 to 7.14 (*p* < 0.01, *d* = 0.56), PBQ-IB (13.04 to 10.27; *p* < 0.01, *d* = 0.27), PBQ-RPA (7.05 to 4.89; *p* < 0.01, *d* = 0.32), ECBQ-EC (4.70 to 4.94, *p* *=* 0.04, *d* = 0.08), and EQ-5D-VAS (56.46 to 65.63; *p* *<* 0.01, *d* = 0.33) were also observed. While a statistically significant interaction between group and time predicted change in PBQ-IFA scores, no difference was detected between T1 and T2 among experimental group participants (3.52 *v.* 3.79, *p* = 0.25, *d* = 0.06) (Table 3).

**Table 2.** Group × time interaction predicting clinical outcomes

Outcome measure	*B*	Std. error	*t*	*p*
EPDS	−2.97	0.46	−6.49	<0.001*
GAD	−2.90	0.49	−5.92	<0.001*
PBQ-IB	−1.41	0.68	−2.08	0.04*
PBQ-RPA	−1.51	0.43	−3.54	<0.001*
PBQ-IFA	−0.66	0.31	−2.15	0.03*
IBQR-SUR	0.17	0.12	1.49	0.14
IBQR-EC	0.12	0.09	1.42	0.16
IBQR-NA	0.07	0.11	0.66	0.51
ECBQ-SUR	0.16	0.22	0.72	0.48
ECBQ-EC	0.42	0.19	2.16	0.04*
ECBQ-NEG	−0.06	0.19	−0.30	0.77
SDQ-TOTAL	0.63	0.99	0.64	0.52
EQ-5D-VAS	7.37	2.12	3.47	<0.001*

EPDS, Edinburgh Postnatal Depression Scale; GAD-7, General Anxiety Disorder-7 Scale; PBQ, Postpartum Bonding Questionnaire; IB, impaired bonding; RPA, rejection and pathological anger; IFA, infant focused anxiety; IBQ-R, Infant Behavior Questionnaire-Revised; ECBQ, Early Childhood Behavioral Questionnaire; SUR, surgency; EC, effortful control; NA, negative affect; SDQ, Strengths and Difficulties Questionnaire; EQ-5D-VAS, EuroQol 5-Dimensional Health Questionnaire Visual Analog Scale.

*Note:* Analyses were conducted using LMMs controlling for workshop facilitator as a fixed effect.

* Statistically significant (*p* < 0.05) mean difference.

**Table 3.** Changes from T1 to T2 for primary and secondary outcome measures

	Experimental group (*N* = 189)						Control group (*N* = 203)					
	Time 1		Time 2				Time 1		Time 2			
	Mean	s.d.	Mean	s.d.	Δmean	*p* value	Mean	s.d.	Mean	s.d.	Δmean	*p* value
EPDS	15.77	5.87	11.22	6.15	−4.55	<0.01*	15.05	4.89	13.49	5.04	−1.56	<0.01*
GAD	11.36	6.08	7.14	6.41	−4.22	<0.01*	11.32	5.06	10.01	5.24	−1.31	<0.01*
PBQ IB	13.04	8.22	10.27	8.62	−2.77	<0.01*	11.79	6.82	10.46	7.02	−1.34	0.01*
PBQ RPA	7.05	5.38	4.89	5.62	−2.16	<0.01*	5.91	4.45	5.26	4.57	−0.65	0.03*
PBQ IFA	3.52	3.71	3.79	3.57	0.27	0.23	3.61	2.99	4.54	2.85	0.93	<0.01*
IBQ-R SUR	4.28	1.31	4.86	1.37	0.58	<0.01*	4.36	1.08	4.76	1.13	0.40	<0.01*
IBQ-R EC	5.14	1.04	5.27	1.11	0.12	0.06	5.24	0.87	5.24	0.90	−0.01	0.94
IBQ-R NA	3.73	1.36	3.90	1.43	0.18	0.03*	3.82	1.13	3.92	1.17	0.10	0.16
ECBQ SUR	5.40	3.26	5.47	3.46	0.07	0.68	5.24	2.55	5.15	2.71	−0.09	0.54
ECBQ NEG	3.31	2.39	3.47	2.58	0.16	0.27	2.94	1.88	3.15	2.02	0.21	0.09
ECBQ EC	4.70	2.56	4.94	2.75	0.24	0.10	5.04	2.01	4.86	2.17	−0.18	0.17
SDQ TOTAL	11.20	11.88	11.80	12.02	0.60	0.20	10.16	9.80	10.29	9.40	0.12	0.47
EQ-5D-VAS	56.46	21.96	65.63	23.34	9.18	<0.01*	56.99	18.72	58.83	19.26	1.84	0.23

EPDS, Edinburgh Postnatal Depression Scale; GAD-7, General Anxiety Disorder-7 Scale; PBQ, Postpartum Bonding Questionnaire; IB, impaired bonding; RPA, rejection and pathological Anger; IFA, infant focused anxiety; IBQ-R, Infant Behavior Questionnaire-Revised; ECBQ, Early Childhood Behavioral Questionnaire; SUR, surgency; EC, effortful control; NA, negative affect; SDQ, Strengths and Difficulties Questionnaire; EQ-5D-VAS, EuroQol 5-Dimensional Health Questionnaire Visual Analog Scale.

*Note:* Analyses were conducted using LMMs controlling for workshop facilitator as a fixed effect, yielding estimated marginal means.

* Statistically significant (*p* < 0.05) mean difference and group × time interaction.

58% of the experimental group *v.* 31% control group had a clinically significant change (>4 points) in EPDS score.

54% of the experimental group *v.* 27% control group had a clinically significant change (>4 points) in GAD-7 score.

Among experimental group participants, 58% reported clinically significant (⩾4-point) decreases in EPDS scores at T2, compared to 31% of control group participants [odds ratio (OR) 3.00, 95% confidence interval (CI) 1.93–4.67, number needed to treat (NNT) = 3.8]. Likewise, 54% of experimental group participants reported a ⩾4-point decrease in GAD-7 scores at T2 *v.* 27% of control group participants (OR 3.20, 95% CI 2.03–5.04, NNT = 3.7).

The largest healthcare costs that accrued between T1 and T2 were attributable to general practitioner use (219 participants with at least one visit). Diagnostic imaging procedures (42 participants) were common but represented highly variable costs. While infrequent (eight participants), overnight hospital admissions also represented sizeable healthcare costs. Over the 12-week course of the study, experimental group participants accrued on average $2076.64 in healthcare costs, while control group participants accrued $4678.78.

**Fig. 2.**
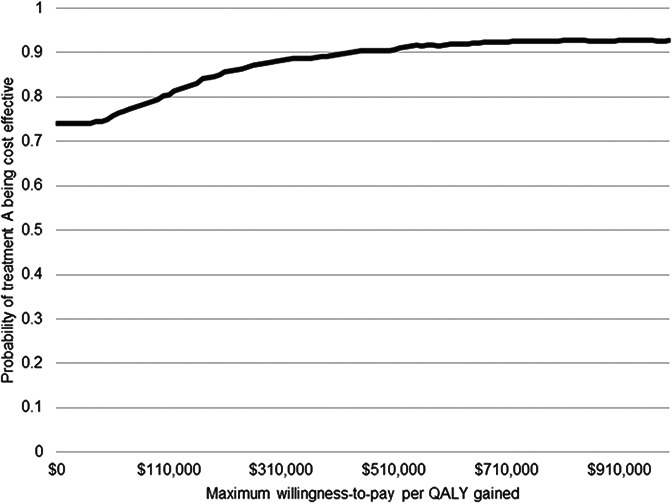
CEAC of treatment across increasing willingness to pay thresholds.

QALYs were calculated for the 12-week time horizon between T1 and T2 for all participants who provided EQ-5D-3L index scores at T2 (experimental group *n* = 150, control group *n* = 179). The mean QALY increase was larger for experimental group participants [0.17 (s.d. = 0.02)] than for the control group [0.16 (s.d. = 0.03)] (the maximum of QALY over 12 weeks is 0.23). This difference was not statistically significant (*t* *=* −1.48, *p* *=* 0.14). However, the 1-day CBT workshop plus TAU achieved similar QALY at lower costs than TAU alone. The probability of the 1-day CBT workshop being cost-effective was 0.74 at a willingness-to-pay threshold of $0 and increased to 0.8 at $100 000 (Fig. 2).

## Discussion

In this RCT of 461 mothers and birthing parents with PPD, a 1-day CBT-based workshop for PPD added to TAU led to statistically and clinically significant reductions in depression and anxiety relative to TAU alone. The workshops also resulted in improvements in the mother–infant relationship and the behavior of toddlers. In addition to its clinical effects, the workshops were cost-saving.

These findings replicate and extend those of an RCT of 1-day CBT-based workshops for PPD delivered online during the COVID-19 pandemic, and provide converging evidence supporting their effectiveness as a low-intensity intervention in stepped-care pathways (NICE, 2020; PCMCH Guidance Document: Perinatal Mental Health, 2021). Indeed, medium effect size improvements were seen in PPD and anxiety in both in-person and online studies, and small effect size increases in the bonding subscale of the PBQ were seen in both interventions. The effectiveness of this 1-day workshop approach to depression treatment is also supported by studies that have examined 1-day interventions for general population samples of adults with depression (Horrell et al., 2014) and/or anxiety (Brown et al., 2000), and adolescents (Brown et al., 2019), as well as other, briefer single-session interventions (Dobias, Schleider, Jans, & Fox, 2021; Schleider et al., 2022). The magnitude of the treatment effects seen are also similar to 1-day workshops in these other groups, as well as longer CBT treatment protocols previously utilized to treat depression in the perinatal period (Sockol, 2015).

In addition to reductions in PPD, in-person and online workshops were both associated with meaningful improvements in anxiety. CBT is a first-line treatment for anxiety in adults (Andrews et al., 2018; Katzman et al., 2014), and a growing body of evidence supports the effectiveness of CBT in those with postpartum anxiety (e.g. Nillni, Mehralizade, Mayer, & Milanovic, 2018). The size of the effect of this intervention on anxiety is comparable to our previous online workshop, as well as existing RCTs of CBT for anxiety (Loughnan et al., 2019; Maguire, Clark, & Wootton, 2018), and suggest that these workshops may be capable of reducing comorbid anxiety in those with PPD.

This intervention also led to some improvement in aspects of the mother–infant relationship including bonding, rejection, and pathological anger, and infant-focused anxiety. These findings replicate those of our previous online RCT (Van Lieshout et al., 2021), as well as other recent trials of CBT for PPD (Amani et al., 2021; Stein et al., 2018; Van Lieshout et al., 2022). Whether these changes are due to improvements in depression or anxiety, or the ways in which mothers interact with their infants are not known. However, while participants reported improvements in the mother–infant relationship, they did not note changes in infant temperament. This is at odds with the findings of the previous online 1-day trial where small but statistically significant increases in surgency were noted. Whether this is due to differences in our sampling frame, sample, or delivery modality (in-person *v.* online) is not known.

Despite the lack of statistically significant changes in infant temperament or the behavior of the participants' older children, improvements in the temperament (effortful control) of their toddlers were reported. Relatively few studies have examined the impact of interventions for PPD on non-infant offspring, even though it is well-known that PPD can have an adverse impact on their emotional and behavioral development. Pre-pandemic studies showed that children exposed to PPD manifest elevated levels of anxiety (Walker, Davis, Al-Sahab, & Tamim, 2013), as well as hyperactivity and concentration difficulties at 2 years of age (Avan, Richter, Ramchandani, Norris, & Stein, 2010). It is not clear why workshops may have had a positive effect on toddlers but not infants or older children. Perhaps changes in toddlers occur more quickly than in infants, or were more easily observable, and/or that the changes occur in temperament, but not child behavioral difficulties. However, replication in future samples is required in order to better understand the potential effects of workshops on offspring.

We found that 1-day CBT-based workshops plus TAU were associated with similar QALYs to TAU alone, but were less expensive. This finding contrasts with a systematic review that found group CBT interventions to be associated with QALY gains but at a cost of £39 875 per QALY gained (Stevenson et al., 2010), well above the NICE-recommended cost-effectiveness threshold of less than £20 000–30 000 (NICE, 2013), suggesting that these interventions may not be cost-effective. However, our cost-effectiveness analyses suggested that 1-day CBT-based workshops for PPD added to TAU may be more effective and less expensive than TAU alone. In this study, the waitlist group also had worse overall health outcomes, though the incremental QALY difference was small. Regardless, these results support demonstrate the potential of the 1-day workshop model to be cost-effective, though further confirmation in future studies would be of value.

While promising, the findings of this study do need to be interpreted in light of its limitations. First, the intervention was delivered by only two expert therapists from a single center and occurred in-person prior to the pandemic. Facilitators of participation in in-person psychotherapies like implementing and clearly communicating safety protocols, conducting sessions outside of hospital settings, the provision of childcare, as well as flexible scheduling should be kept in mind in order to optimize participation in workshops delivered face-to-face (Andrejek et al., 2021).

While some prefer to attend interventions in-person, this modality may now be less popular than online delivery for some mothers and birthing parents. Online treatments have gained popularity since the COVID-19 pandemic for their increased accessibility (Simpson, Richardson, Pietrabissa, Castelnuovo, & Reid, 2021), families and healthcare providers have expressed significant interest in using telemedicine for PPD (Singla et al., 2020), and online therapeutic alliances have been rated as strongly as in in-person settings (Simpson & Reid, 2014).

While full-day workshops are effective, we should acknowledge that some may prefer the intervention be delivered in two half-days. However, in our online trial, just 10 of 161 participants in the experimental group (6%) expressed a preference that it be delivered differently (e.g. in half-days). Of these, 155 (96%) remained online for the entire session and 140 (87%) indicated that they were very satisfied with the full-day workshop (Van Lieshout et al., 2021).

While facilitated interventions are often preferred by individuals with PPD over other self-guided options (Goodman, 2009), future research should focus on exploring alternative delivery modalities, along with an examination of the potential moderators (e.g. therapist type, participant PPD severity, delivery in a single-day *v.* two half-days) and mediators (intervention fidelity, cognitive, and/or behavioral skill use) of intervention effectiveness. In the interim, asking those with PPD what they feel might work best for them is likely the optimal approach.

This study also took place in Canada where healthcare is universally available and so our findings may not generalize to all settings. Attrition was also higher in the experimental group in this study, but this may be due to the anticipation of those on the waitlist to receive the workshop. This is a consistently observed effect in studies of 1-day CBT-based workshops (Horrell et al., 2014; Van Lieshout et al., 2021). While the workshops did produce clinically significant improvements in PPD and anxiety, we did not conduct structured clinical interviews and so participants may not have had syndromal MDD or GAD, nor did they necessarily experience remission. However, since PPD is commonly seen as a continuous construct and the improvements observed in this and our previous study are clinically meaningful, the intervention may still have clinical utility. It is also important to note that all outcomes were self-reported by participants and so improvements in the mother–infant relationship and offspring outcomes may be contributed to by maternal symptom improvement. Moreover, it is possible that spontaneous remission could have contributed to our findings. Indeed, in previous trials of those with PPD, approximately 25% of participants were thought to have experienced spontaneous recovery (Li et al., 2022; Sockol, 2015, 2018). We feel that we should also emphasize that while we chose to assess our outcomes at a single timepoint (3 months post-intervention) to try to optimize the comparability of our post-treatment findings to trials of full courses of structured psychotherapies, we did not assess participants immediately post-treatment (the day after the workshop). Because our study was powered to detect a moderate (*d* = 0.3) treatment effect in our primary outcome of interest (EPDS), it had less statistical power than might have been necessary to detect an equivalent effect in those outcomes that applied only to those with toddlers. Future studies may more comprehensively investigate the effect of CBT treatment on these outcomes with a larger sample size, specifically recruiting participants with children aged 2–3 years. Finally, we utilized a waitlist control design which may have inflated the size of the effects observed and prevented us from assessing the longer-term impact of the intervention.

Those focused on the mental health of mothers and birthing parents are in urgent need of safe, efficient, economical, evidence-based, and scalable means of treating PPD. One-day CBT-based workshops may be an effective treatment for PPD delivered in-person or online and has significant potential for scaling and task-shifting. It is also consistent with mothers' preferences and the proportionate universalism approach to public mental health utilized around the world, which attempts to address the whole population while providing additional (selective/targeted/indicated) support for groups at risk. These workshops may also represent a perinatal-specific option that could be integrated into stepped care approaches at reasonable cost. If successful, this intervention could provide the scope to deliver treatment to large numbers of individuals with PPD, an important goal given the staggering cost of this disorder and the importance of parental mental health to children, families, and society.

## Supplementary material

For supplementary material accompanying this paper visit https://doi.org/10.1017/S0033291723000454. click here to view supplementary material
