# Supplementary material for: In-person 1-day cognitive behavioral therapy-based workshops for postpartum depression: a randomized controlled trial
Source: Psychol Med. 2023 Mar 7;53(14):6888–98. doi: 10.1017/S0033291723000454 (PMC10600825; doi:10.1017/S0033291723000454)
Supplement: Supplementary file 1 [file S0033291723000454sup001.pdf]

Supplemental Table 1

Valuation of Service Unit Costs

Service	Unit	Cost
Emergency Department visit (*Emergency Medicine: Niagara Health*, n.d.)	Per visit	$ 2,587.80
Overnight (or longer) stay in hospital ("Patient Cost Estimator | Canadian Institute for Health Information," n.d.)	Per visit	$ 12,835.00
Diagnostic Procedure – ultrasound (*Schedule of Facility Fees: For Independent Health Facilities*, 2020)	Per test	$ 125.80
Diagnostic Procedure – X-Ray (*Schedule of Facility Fees: For Independent Health Facilities*, 2020)	Per test	$ 50.82
Diagnostic Procedure – CT ("Ontario, OR CT Scan Cost Average," n.d.)	Per test	$ 876.00
GP/Family Doctor (Government of Ontario, n.d.)	Per visit	$ 45.90
Physiotherapist ("Business and Personal Resources – Ontario Physiotherapy Association," n.d.)	Per hour	$ 70.64
Occupational Therapist ("Occupational Therapist (OT) in Canada | Labour Market Facts and Figures - Job Bank," n.d.)	Per hour	$ 43.08
Speech Language Pathologist (*Recommended Fee Schedule: Speech-Language Pathologists & Speech-Language Pathology Services*, 2020)	Per hour	$ 203.00
Nutritionist and Dietician (*The Dietitian Workforce in Ontario Primary Health Care Survey Report*, 2018)	Per hour	$ 34.30
Chiropractor(*OCA Recommended Service Codes and Fee Schedule*, 2019)	Per hour	$ 294.00
Social Worker/Other counsellor ("A Snapshot of Social Work in Ontario: Key Indicators for Sustaining a Critical Profession," n.d.)	Per hour	$ 47.13
Massage Therapist ("Registered Massage Therapists' Association of Ontario - Services and Fees," n.d.)	Per hour	$ 91.00
